# Attitudes and Intentions toward COVID-19 Vaccination among Spanish Adults: A Descriptive Cross-Sectional Study

**DOI:** 10.3390/vaccines9101135

**Published:** 2021-10-04

**Authors:** Diego Gabriel Mosteiro-Miguéns, Daniel De Bernardo Roca, Eva María Domínguez-Martís, Natalia Vieito-Pérez, Pilar Álvarez-Padín, Silvia Novío

**Affiliations:** 1 Galician Public Healthcare Service, Healthcare Centre of Concepción Arenal, C/Santiago León de Caracas 12, 15701 A Coruña, Spain; diego.gabriel.mosteiro.miguens@sergas.es (D.G.M.-M.); eva.maria.dominguez.martis@sergas.es (E.M.D.-M.); 2 Galician Public Healthcare Service, Healthcare Centre of A Bandeira, Pza. Camilo José Cela s/n, 36570 Pontevedra, Spain; daniel.de.bernardo.roca@sergas.es; 3 Galician Public Healthcare Service, University Hospital Complex of Santiago de Compostela (CHUS), C/ Choupana s/n, 15706 A Coruña, Spain; natalia.vieito.perez@sergas.es; 4 Castilla y León Public Healthcare Service, University Hospital of Burgos, Avda Islas Baleares 3, 09006 Burgos, Spain; papadin@saludcastillayleon.es; 5 Department of Psiquiatry, Radiology, Public Health, Nursing and Medicine, University of Santiago de Compostela, C/ San Francisco s/n, 15782 A Coruña, Spain

**Keywords:** attitude, COVID-19, intention, prevention, SARS-CoV-2, vaccines

## Abstract

Vaccination against SARS-CoV-2 is postulated as the most effective measure to control the COVID-19 pandemic. However, the use of other protection measures is necessary to efficiently combat the spread of the virus. The aim of the present study was to determine the attitudes and intentions toward COVID-19 vaccination among non-regular social media users in Spain and to analyze how these factors could condition the acceptance of other personal protective measures once an individual has received the COVID-19 vaccine. A cross-sectional design was used in this work. In total, 719 subjects, ≥18 years old and of both sexes, were recruited from primary public healthcare centers to self-complete a questionnaire between March and April 2021. The majority of participants had a positive attitude toward vaccination and showed high levels of intention to be vaccinated. Likewise, except those participants who considered the vaccine to be the most effective measure to fight the COVID-19 pandemic, the rest of the participants highlighted the importance of continuing to limit social interactions and/or wearing masks even after being vaccinated. Since vaccination can create a perception of total immunity against SARS-CoV-2, it is necessary that healthcare staff organize effective awareness campaigns on the importance of maintaining personal protective measures until vaccination coverage is greater.

## 1. Introduction

The severe acute respiratory syndrome coronavirus 2 (SARS-CoV-2) pandemic is one of the most important health challenges of the last century [1] and is producing significant psychological, social and economic consequences [2,3]. To date, there is still no definitive treatment for this viral respiratory infection, so prevention is essential [4].

Since the beginning of the pandemic, all hopes for definitive control of the virus, especially in at-risk groups [5,6,7,8], were pinned on the development of a vaccine against SARS-CoV-2 [9]. This is because in addition to the individual protection that it offers, a vaccine’s effectiveness is based on its collective effects [10], which depend on the number of people who are willing to be vaccinated, among other factors. Since approximately 70% of the population [11,12] must be vaccinated to achieve the maximum efficacy of this biomedical strategy [11,12,13], one of the major concerns among health authorities, even before the approval of the vaccine by the Food and Drug Administration (FDA) or the European Medicines Agency (EMA), has been vaccine hesitancy [14]. This concern gave rise to a considerable number of studies seeking to determine the attitudes toward COVID-19 vaccination and the intention to receive a vaccine against SARS-CoV-2 and/or the factors influencing such decisions [15,16].

There is no doubt about the usefulness of the vaccine; however, in addition to vaccination other hygiene measures, such as surface disinfection practices [17], hygienic sanitary measures such as hand hygiene [18] and the use of a hydroalcoholic solution [19], as well as physical and social distancing [20,21] and/or wearing masks [22,23], are necessary. Even though all of these measures have been useful since the beginning of the pandemic, their efficacy could be underestimated due to the appearance of vaccines, which are considered to be the most effective measure for achieving definite control of the virus [24].

Based on the aforementioned data, educational interventions provided by healthcare professionals are required, as a lack of information or misinformation could endanger control of the pandemic [25]. Thus, the objective of this study was to determine the attitudes and intentions toward COVID-19 vaccination among Spanish adults, and more specifically among non-regular social media users and analyze how these factors could condition the acceptance of other personal protective measures once an individual has received the vaccine.

## 2. Materials and Methods

### 2.1. Design

A cross-sectional, descriptive study was carried out.

### 2.2. Setting and Participants

The study was conducted in the Health Area of Santiago de Compostela and Barbanza, one of the 7 Galician Health Areas. A sample group of 719 subjects was recruited from primary public healthcare centers in this health area by general service, nursing and medical staff who were willing to participate in the study.

The investigation included patients of either sex, 18 years of age or older, who went to the primary public healthcare centers for any health reason. Patient companions, individuals who could not read or write in Spanish and those who had already received one dose of the COVID-19 vaccine were excluded from the study. All eligible patients within the period of the study were approached.

The size of the study population was 350,000 at the time of the research. Keeping the expected frequency of all variables at 50%, the most desirable sample size using a 95% confidence interval was determined to be 599. However, after 15% inflation and rounding, the final desired sample size was determined to be 689.

### 2.3. Questionnaire Design and Data Collection

The questionnaire was designed according to the advice of healthcare professionals based on a literature review [26]. A pilot study was conducted with 15 people who did not participate in the final study in order to evaluate the clarity and ease of understanding of the items, as well as the filling time, of the questionnaire. The pilot participants reported full comprehension of the questions and ease in completing the questionnaire, so only minimal changes were made following the pilot study.

The questionnaire consists of 20 items structured into four sections (Supplementary Materials Table S1). The first and second sections included 4 and 3 questions about sociodemographic characteristics (age, sex, education level and occupation) and clinical features, respectively. The third section measures participants’ attitudes towards the COVID-19 vaccine and their intentions of receiving the COVID-19 vaccination using 8 closed-ended questions and one question with a five-point Likert scale (1 = no confidence to 5 = high confidence). The last section used four questions with a five-point Likert scale (1 = strongly agree to 5 = strongly disagree) to determine the intended behaviors of the participants related to social distancing and wearing masks in different situations after receiving the COVID-19 vaccine.

The questionnaires, which were distributed in person, were anonymous and self-completed between March and April 2021. Participants were free to omit any questions they did not want to answer. No incentive was offered for completing the questionnaire. In each primary public health care center, a person was assigned to collect and safeguard the questionnaires once they were filled in.

### 2.4. Ethical and Legal Considerations

The protocol of the study was evaluated by the Research Ethics Committee of Santiago-Lugo (registration code 2021/054).

After explaining the procedure and objective of the investigation, we obtained the participants’ consent and explained that their participation was completely voluntary. Pursuant to the Declaration of Helsinki and the Data Protection Act (Organic Law 3/2018), data confidentiality was guaranteed at all times.

### 2.5. Statistial Analysis

The results are presented as a number and percentage or mean and standard deviation. Numerical (Kolmogorov–Smirnov test; skewness; kurtosis; and the relationships between the mean, median and mode) and visual (Q–Q plot) methods were used to test the normality of the data.

Bivariate analysis was performed using ANOVA and Student’s *t*-tests for continuous variables and chi-square tests for categorical variables. Significance between multiple groups was determined using Tukey’s post hoc analysis. Throughout the study, a *p*-value less than 0.05 was considered significant. GNU PSPP 0.8.4 (Free Software Foundation Inc., Boston, MA, USA) and Epidat version 4.2 (Xunta de Galicia, Santiago de Compostela, Spain) were used for statistical processing of the data.

## 3. Results

### 3.1. Description of Sample

A total of 719 subjects chose to participate in the study. However, 33 were excluded because they were less than 18 years old (*n* = 2) or had already received one dose of the COVID-19 vaccine (*n*= 31).

Table 1 shows the sociodemographic and clinical characteristics of the participants. The sample was composed primarily of women (62%), with a mean age of 52.8 years and no relevant diseases, except for the presence of hypertension. One-third of the participants had completed secondary education, with 20% having studied at university, although almost half of the participants referred to not working at the time, they completed the questionnaire.

### 3.2. Attitudes toward COVID-19 Vaccine and Intention of Receiving COVID-19 Vaccination

The majority of the participants considered the vaccine to be the most effective measure for combatting the COVID-19 pandemic, especially in combination with other protective measures (item 8). Furthermore, the respondents were very willing to receive the vaccine (item 13) due to the confidence that it gave them (item 14) and its perceived level of safety (item 11). The youngest participants, those with the highest level of education, and those who had been vaccinated against the flu presented the most positive attitudes toward the vaccine and the highest intention of being vaccinated (Table 2 and Table 3).

More than 90% of the participants considered informative briefings about the vaccine against SARS-CoV-2 to be useful (item 15), whether such briefings were conveyed by a doctor or by a nurse. However, only half of the participants said that they had researched information about the vaccine in the last month (item 9), mainly through internet. Significant differences were not found in educational needs according to the gender, age or level of education among the participants (Table 2 and Table 3).

When the participants were asked if they believed that they should receive the vaccine, more than 95% agreed (item 12). Female participants, the oldest participants and who had been vaccinated against the flu in the last year were significantly more aware of the need to have the vaccine (Table 2 and Table 3). On the contrary, significant differences were not found in belief that the vaccine is necessary based on whether the participant did or did not belong to an at-risk population (*p* = 0.251).

### 3.3. Personal Protective Measures after Vaccination

The majority of the participants would not increase their social interactions even after being vaccinated (item 17), neither would they reduce the use of a mask with their regular contacts (items 18) or in closed public areas (item 19); they would consider using a mask less often only in open areas (item 20) (Table 2). However, it should be noted that when determining how the attitudes toward vaccination and/or the intention of receiving a COVID-19 vaccination could influence the use of personal protective measures after vaccination, the following significant differences were observed: i) the respondents who considered the vaccine to be the most effective measure to fight the COVID-19 pandemic noted that they would increase their social interactions in the short term and would use a mask less often, in comparison with those who did not consider the vaccine to be the most useful measure or thought that a vaccine would be a useful measure in combination with other preventive measures; ii) the respondents willing to receive the vaccine were found to be more reluctant to reduce the use of a mask in closed public places than those who were not willing to receive a vaccine (Table 4).

In general, male participants, the youngest participants and those with the lowest level of education were the most strongly in favor of reducing preventive measures against SARS-CoV-2 after being vaccinated (Table 2 and Table 3).

## 4. Discussion

The results of the present study reveal that the attitudes and intentions of the non-regular social media users in Spain toward vaccination are favorable. However, this confidence is high enough that it could compromise the maintenance of other individual protective measures, especially among those who consider the vaccine to be the most effective measure to fight the pandemic. To our knowledge, this is the first study on determining the intention of Spanish adults to be vaccinated that obtained information using in-person questionnaires, which allowed us to reach a population group for which we had no information, as members of this group are not regular users of social media.

Even before the current pandemic, both the emergence of virulent pathogens and vaccine hesitancy were identified by the World Health Organization as potential threats to global health [27]. Different studies have evaluated the intentions of the general population toward COVID-19 vaccination, indicating considerable differences between countries. The highest intention of vaccination during the first year of the pandemic was observed in China (91.3%) [28], while the countries with the worst statistics on vaccination intention were Nigeria (29%) [29] and Kuwait (23.6 %) [30]. Spain [31], France, Italy [32,33], the United Kingdom [34,35] and several other countries [36,37,38,39,40,41] instead occupy intermediate positions. In Spain, current data on vaccination intention show variable results, with percentages that range from approximately 75% [31,37] to 48.3% [42]. As it is estimated that vaccination coverage should be approximately around 70% to achieve maximum effectiveness [11,12,13], the Spanish estimates could suggest the inability of our country to reach the desired herd immunity threshold.

During the pandemic in Spain, fake news on COVID-19 has been published on social media and instant messaging apps [43,44], the main media outlets used by the participants of the present study to remain informed about vaccination (item 10). However, this issue does not seem to have had a negative impact on the intention to be vaccinated in our country.

Apart from the study carried out by Rodríguez-Blanco et al. [42], the rest of the studies performed using a representative sample of the general population at a national level [37], as well as samples of the population originating from different Autonomous Communities in Spain (Catalonia [31]) and Galicia [current study]), show that the intention to be vaccinated has remained consistently high, as the vaccine has given the population significant confidence. This finding contrasts sharply with the results observed in other countries such as Italy, the UK and China [30,45,46], where in addition to a decrease in the intention to be vaccinated, there is evidence of an increase in a refusal to be vaccinated.

Among the different reasons referred to by the population in not accepting the vaccine, the main concerns include a fear of long-lasting health problems or side effects [38,47,48,49,50], antivaccine beliefs [38,47,51,52,53] and the need for more information [38,53], among others [38,47,49,54,55]. Among these reasons, a lack of vaccine safety was noted to be the main reason to refuse the vaccine [38,41,47,56,57,58], which could be due to the population’s lack of trust in the speed at which the vaccines were developed [59,60], as well as contradictory information given by politicians over time [42]. As stated by more than 90% of the sample in the current study (item 15), this matter highlights the need for healthcare professionals to organize informative briefings about the vaccine against SARS-CoV-2 to highlight that the vaccine is not only effective but also safe. In Spain, doctor–nurse collaboration is essential to carry out behavioral and biomedical interventions in Primary Health Care, such as vaccination campaigns, in which these healthcare professionals can not only contribute to raising awareness about the importance of becoming vaccinated but also aid in advising, diagnosing and/or monitoring potential side effects [61]. Moreover, according to the results of our study, such briefings should be mainly aimed at people with low levels of education, as such individuals showed the lowest intention to be vaccinated, as already mentioned by other authors [38,62,63].

We have yet to fully control the pandemic, as the appearance of new highly transmissible SARS-CoV-2 variants has contributed to the occurrence of new outbreaks; such outbreaks highlight the fact that this is a very contagious virus [64,65,66,67]. Even though the vaccines are of great help in protecting people against SARS-CoV-2, these vaccines may not be effective against all the variants. Therefore, we should not mistakenly believe that the vaccine alone will control the pandemic [68,69]. Even when a person is vaccinated, new variants circulating in the community can cause COVID-19. This matter highlights the need to inform the population about the importance of using public health measures that are highly effective, such as wearing masks and engaging in social distancing, even when an individual is already vaccinated [70,71]. Indeed, in the present study, those who considered the vaccine to be the most effective measure to fight the COVID-19 pandemic also said they would increase their social interactions in the short term and would use a mask less often in the presence of their regular contacts, as well as in closed public and open areas. The effectiveness of personal protective measures has been a controversial subject over the course of the pandemic. Even though the Spanish political health authorities initially advised against wearing a mask [72], as reported by traditional communication media [73], subsequent recommendations were very different in various countries [74,75,76,77,78], which may have led to doubts regarding the true usefulness of such measures [79].

In Spain, an insufficient number of vaccines made it necessary to establish a vaccination strategy against COVID-19 that prioritized at-risk groups, in addition to meeting other ethical principles [8]. Thus, after health and social health workers were vaccinated, the following groups were prioritized: elderly people aged >80 followed by patients with hypertension, diabetes mellitus, cardiovascular diseases, chronic obstructive pulmonary disease, stroke, stage 3–5 chronic renal failure, hepatic cirrhosis, neoplasia and immunosuppression [5,6,7,8]. Even though these population groups (especially those above the age of 80) had the highest rates of hospitalization and mortality [80,81,82], in the current study, significant differences were not found between at-risk groups and other groups regarding belief in whether the vaccine is necessary.

In contrast with other studies carried out among Spanish adults to determine the intention to be vaccinated, this is the only study to date that obtained the information in person, which allowed us to acquire information on populations who are not regular users of social media. However, these results must be interpreted with caution due to some limitations. First, as the participants filled in the questionnaires themselves, there may have been some self-reporting bias. Second, we asked the participants about their intent to be vaccinated at a time when vaccination was already available. Thus, it is possible that the answers from some of the participants may have been influenced by the opinions of other people who had already been vaccinated. Third, according to the method used to obtain the information for the current study, the sample consists of non-regular social media users, which is not necessarily representative either of them or of the general population.

## 5. Conclusions

Since vaccination can create a perception of total immunity against SARS-CoV-2, partly due to a lack of hope in fighting the different stages of COVID-19 in the absence of effective strategies to combat the disease [83], it is necessary that healthcare professionals organize effective awareness campaigns aimed at Spanish adults, especially to non-regular social media users, on the importance of maintaining personal protective measures until vaccination is more widespread.

## Figures and Tables

**Table 1:** Sociodemographic and clinical characteristics of the study’s participants (*n* = 686).

	N (%)
**Age, mean (SD)**	52.77 (15.9)
**Sex, *n* (%)**	
Male	239 (34.8)
Female	425 (62.0)
**Level of education,** ***n*** **(%)**	
Without studies	25 (3.6)
Primary	264 (38.5)
Secondary	252 (36.7)
Graduate or above	140 (20.4)
**Occupation,** ***n*** **(%)**	
Unemployed	134 (19.5)
Retired	159 (23.2)
Livestock/Agriculture	32 (4.7)
Transport sector	20 (2.9)
Teaching	30 (4.4)
Food industry	33 (4.8)
Catering industry	33 (4.8)
Other	239 (34.8)
**Personal medical history** **,** ***n*** **(%)**	
Severe allergies to vaccines	12 (1.8)
Drug allergy	59 (8.6)
Previous infection by SARS-CoV-2	24 (4.8)
COPD	17 (2.5)
Asthma	55 (8.0)
Hypertension	142 (20.7)
Myocardial infarction	13 (1.9)
Heart failure	26 (3.8)
Heart valve disease	34 (5.0)
Oncological disease	27 (3.9)
Immunosuppression	11 (1.6)
HIV infection / AIDS	2 (0.3)
Diabetes	42 (6.1)
Neurological disorders	12 (1.8)
Pregnancy	22 (3.2)
Lactation	5 (0.7)
Other	59 (8.6)
**Vaccinated against the flu in 2020** **, ** ***n*** **(%)**	283 (41.8)

Abbreviations: AIDS, acquired immunodeficiency syndrome; COPD, chronic obstructive pulmonary disease; HIV, human immunodeficiency virus; SARS-CoV-2, severe acute respiratory syndrome coronavirus 2; SD, standard deviation.

**Table 2:** Attitudes toward the COVID-19 vaccine, intentions of receiving COVID-19 vaccination and personal protective measures after vaccination according to age.

	Total	Age (Years)				
		18–39	40–59	60–79	≥80	*p*
**Q8. Is the COVID-19 vaccine the most effective measure to fight the pandemic?**						
No	15 (2.4)	5 (4.6)	8 (2.5)	2 (1.5)	0	0.000
Yes	142 (22.3)	17 (15.6)	58 (18.4)	40 (30.8)	17 (39.5)	
Yes, in combination with other protection measures	480 (75.4)	87 (79.8)	249 (79.1)	88 (67.7)	26 (60.5)	
**Q9. Have you looked for information on the COVID-19 vaccine in the last month?**						
No	312 (51.6)	54 (46.6)	142 (48.1)	72 (60.5)	22 (55)	0.084
Yes ^a^	293 (48.4)	62 (53.5)	153 (51.9)	47 (39.5)	18 (45)	
**Q11. Which of the following COVID-19 vaccines gives you the greatest security?**						
Pfizer–BioNTech	202 (41.3)	34 (38.6)	96 (39.2)	46 (48.9)	13 (39.4)	0.255
Moderna	21 (4.3)	2 (2.3)	11 (4.5)	4 (4.3)	2 (6.1)	
Both	234 (47.9)	47 (53.4)	123 (50.2)	37 (39.4)	14 (42.4)	
None	29 (5.9)	5 (5.7)	12 (4.9)	7 (7.5)	4 (12.1)	
Other	3 (0.6)	0	3 (1.2)	0	0	
**Q12. According your knowledge, do you believe that you should receive the COVID-19 vaccine?**						
No	27 (4.8)	10 (10.9)	12 (4.4)	5 (4.2)	0	0.008
Yes	531 (95.2)	82 (89.1)	261 (95.6)	114 (95.8)	40 (100)	
**Q13. Would you get the COVID-19 vaccine?**						
No	59 (10.2)	10 (9.3)	25 (8.6)	18 (15.5)	4 (11.4)	0.210
Yes	520 (89.8)	98 (90.7)	266 (91.4)	98 (84.5)	31 (88.6)	
**Q14. How much trust do you put in the vaccine?**						
Not confident at all	21 (3.7)	2 (1.9)	13 (4.4)	6 (5.7)	0	0.015
Slightly confident	35 (6.1)	11 (10.3)	13 (4.4)	8 (7.6)	1 (3.1)	
Somewhat confident	153 (26.8)	36 (33.6)	73 (24.9)	26 (24.5)	9 (28.1)	
Fairly confident	152 (26.7)	33 (30.8)	89 (30.4)	17 (16.0)	7 (21.9)	
Completely confident	209 (36.7)	25 (23.4)	105 (35.8)	49 (46.2)	15 (46.9)	
**Q15. Do you consider the informative briefings about the COVID-19 vaccine organized by health centre staff as useful?**						
No	53 (8.9)	8 (7.5)	26 (8.9)	14 (10.9)	5 (13.2)	0.165
Yes ^b^	544 (91.1)	99 (92.5)	266 (91.1)	114 (89.1)	33 (86.8)	
**If I was vaccinated…**						
**Q17. I would increase my social interactions in the short term**						
Strongly agree	55 (9.4)	9 (8.1)	26 (9)	15 (12.7)	3 (9.1)	0.143
Agree	141(24.1)	24 (21.6)	66 (22.8)	33 (28.0)	10 (30.3)	
Neither agree nor disagree	215 (36.8)	47 (42.3)	113 (39.1)	35 (29.7)	9 (27.3)	
Disagree	141 (24.1)	28 (25.2)	70 (24.2)	23 (19.5)	11 (33.3)	
Strongly disagree	32 (5.5)	3 (2.7)	14 (4.8)	12 (10.2)	0	
**Q18. I would consider it reasonable to reduce the use of a mask with my regular contacts (friends, parents…)**						
Strongly agree	67 (11.3)	11 (9.6)	26 (9.1)	15 (12.7)	7 (18.9)	0.026
Agree	100 (16.9)	27 (23.5)	52 (18.1)	14 (11.9)	1(2.7)	
Neither agree nor disagree	97 (16.4)	23 (20)	44 (15.3)	18 (15.3)	8 (21.6)	
Disagree	204 (34.5)	33 (28.7)	113 (39.4)	41 (34.8)	12 (32.4)	
Strongly disagree	123 (20.8)	21 (18.3)	52 (18.1)	30 (25.4)	9 (24.3)	
**Q19. I would consider it reasonable to reduce the use of a mask in closed public areas.**						
Strongly agree	60 (10.2)	6 (5.2)	34 (11.6)	10 (8.8)	3 (8.6)	0.380
Agree	29 (4.9)	6 (5.2)	12 (4.1)	5 (4.4)	4 (11.4)	
Neither agree nor disagree	40 (6.8)	12 (10.3)	17 (5.8)	8 (7.0)	2 (5.7)	
Disagree	208 (35.3)	36 (31.0)	113 (38.7)	41 (36.0)	12 (34.3)	
Strongly disagree	252 (42.8)	56 (48.3)	116 (39.7)	50 (43.9)	14 (40)	
**Q20. I would consider it reasonable to reduce the use of a mask in open areas**						
Strongly agree	116 (19.5)	23 (19.8)	54 (18.1)	21 (19.1)	7 (20.6)	0.856
Agree	147(24.8)	29 (25)	80 (26.9)	24 (21.8)	5 (14.7)	
Neither agree nor disagree	149 (25.1)	27 (23.3)	80 (26.9)	24 (21.8)	9 (26.5)	
Disagree	115 (19.4)	25 (21.6)	55 (18.5)	25 (22.7)	8 (23.5)	
Strongly disagree	67 (11.3)	12 (10.3)	29 (9.7)	16 (14.6)	5 (14.7)	

Statistical significance (*p* < 0.05) was determined by ANOVA and Student’s t-tests. Abbreviations: Q, question. ^a^ The information about the COVID-19 vaccine was obtained from television (*n* = 146, 49.8%), radio (*n* = 48, 16.4%), print media (*n* = 76, 25.9%), internet (*n* = 214, 73.0%), healthcare professionals working in primary healthcare centers (*n* = 65, 22.2%), and other (*n* = 13, 4.4%). ^b^ The preferred healthcare professional to organize the informative briefings about the COVID-19 vaccine was: nurse (*n* = 43, 7.9%), doctor (*n* = 145, 26.7%), nurse or doctor (*n* = 250, 46.0%), indifferent (*n* = 147, 27.0%) and other (*n* = 2, 0.4%).

**Table 3:** Attitudes toward the COVID-19 vaccine, intentions of receiving COVID-19 vaccination and personal protective measures after vaccination according to sex, level of education and flu-vaccine history.

	Sex			Level of Education				Vaccinated against the Flu in 2020		
	Male	Female	*p*	Primary or Lower	Secondary	Graduate or Above	*p*	No	Yes	*p*
**Q8. Is the COVID-19 vaccine the most effective measure to fight the pandemic?**										
No	9 (4.1)	6 (1.5)	0.064	8 (3.0)	4 (1.7)	3 (2.3)	0.021	14 (3.8)	1 (0.4)	0.000
Yes	53 (24.2)	82 (20.6)		75 (28.2)	40 (17.0)	25 (19.1)		65 (17.7)	73 (27.8)	
Yes, in combination with other protective measures	157 (71.7)	310 (77.9)		183 (68.8)	192 (81.4)	103 (78.6)		288 (78.5)	189 (71.9)	
**Q9. Have you looked for information about the COVID-19 vaccine in the last month?**										
No	123 (56.4)	182 (49.2)	0.054	159 (66.8)	117 (50.7)	34 (25.6)	0.000	173 (49.2)	136 (55.3)	0.082
Yes	95 (43.6)	188 (50.8)		79 (33.2)	114 (49.4)	99 (74.4)		179 (50.9)	110 (44.7)	
**Q11. Which of the following COVID-19 vaccines gives you the greatest security?**										
Pfizer–BioNTech	62 (38.8)	134 (42.8)	0.551	80 (44.4)	70 (37.8)	51 (42.5)	0.297	111 (38.1)	87 (45.3)	0.627
Moderna	6 (3.8)	12 (3.8)		9 (5)	9 (4.9)	3 (2.5)		14 (4.8)	7 (3.7)	
Both	79 (49.4)	152(48.6)		77 (42.8)	97 (52.4)	60 (50)		146 (50.2)	86 (44.8)	
None	11 (6.9)	14 (4.5)		13 (7.2)	9 (4.9)	4 (3.3)		18 (6.2)	11 (5.7)	
Other	2 (1.3)	1 (0.3)		1 (0.6)	0	2 (1.7)		2 (0.7)	1 (0.5)	
**Q12. According your knowledge, do you believe that you should receive the COVID-19 vaccine?**										
No	16 (8.2)	11 (3.2)	0.014	7 (3.1)	11 (5.5)	8 (6.4)	0.300	25 (8.0)	2 (0.8)	0.000
Yes	180 (91.8)	333 (96.8)		221 (96.9)	189 (94.5)	118 (93.7)		288 (92.0)	236 (99.2)	
**Q13. Would you get the COVID-19 vaccine?**										
No	24 (11.7)	31 (8.6)	0.241	32 (14.8)	18 (7.9)	8 (6.2)	0.013	36 (10.8)	22 (9.1)	0.506
Yes	181 (88.3)	328 (91.4)		185 (85.3)	211 (92.1)	122 (93.9)		298 (89.2)	220 (90.9)	
**Q14. How much trust do you put in the vaccine?**										
Not confident at all	6 (3.1)	13 (3.6)	0.857	11 (5.1)	6 (2.7)	4 (3.1)	0.065	15 (4.5)	6 (2.5)	0.100
Slightly confident	13 (6.7)	21 (5.8)		13 (6.1)	13 (5.8)	9 (7.0)		24 (7.2)	11 (4.7)	
Somewhat confident	47 (24.2)	100 (27.7)		65 (30.2)	63 (28.1)	24 (18.6)		97 (29.2)	55 (23.3)	
Fairly confident	52 (26.8)	99 (27.4)		42 (19.5)	69 (30.8)	40 (31.0)		86 (25.9)	65 (27.5)	
Completely confident	76 (39.2)	128 (35.5)		84 (39.1)	73 (32.6)	52 (40.3)		110 (33.1)	99 (42.0)	
**Q15. Do you consider the informative briefings about the COVID-19 vaccine organized by health centre staff as useful?**										
No	21 (10.1)	31 (8.4)	0.482	23 (9.7)	15 (6.8)	13 (9.7)	0.479	30 (8.8)	23 (9.1)	0.901
Yes	187 (89.9)	340 (91.6)		215 (90.3)	206 (93.2)	121 (90.3)		311 (91.2)	230 (90.9)	
**If I was vaccinated…**										
**Q17. I would increase my social interactions in the short term**										
Strongly agree	29 (14.3)	24 (6.6)	0.006	29 (12.7)	16 (7.3)	10 (7.6)	0.095	25 (7.2)	30 (12.9)	0.112
Agree	50 (24.6)	86 (23.6)		65 (28.5)	45 (20.5)	31 (23.5)		79 (22.7)	61 (26.2)	
Neither agree nor disagree	76 (37.4)	134 (36.8)		78 (34.2)	89 (40.5)	47 (35.6)		136 (39.1)	77 (33.1)	
Disagree	35 (17.2)	101 (27.8)		49 (21.5)	54 (24.6)	36 (27.3)		87 (25.0)	54 (23.2)	
Strongly disagree	13 (6.4)	19 (5.2)		7 (3.1)	16 (7.3)	8 (6.1)		21 (6.0)	11 (4.7)	
**Q18. I would consider it reasonable to reduce the use of a mask with my regular contacts (friends, parents…)**										
Strongly agree	25 (12.3)	40 (10.8)	0.068	33 (14.7)	24 (10.3)	9 (6.9)	0.322	36 (10.4)	31 (12.8)	0.166
Agree	42 (20.7)	55 (14.9)		36 (16.1)	37 (16.0)	27 (20.6)		69 (20)	30 (12.4)	
Neither agree nor disagree	41 (20.2)	56 (15.1)		30 (13.4)	45 (19.4)	22 (16.8)		56 (16.2)	38 (15.7)	
Disagree	62 (30.5)	137 (37.0)		82 (36.6)	77 (33.2)	45 (34.4)		114 (33.0)	90 (37.2)	
Strongly disagree	33 (16.3)	82 (22.2)		43 (19.2)	49 (21.1)	28 (21.4)		70 (20.3)	53 (21.9)	
**Q19. I would consider it reasonable to reduce the use of a mask in closed public areas**										
Strongly agree	20 (10.0)	39 (10.5)	0.046	29 (13.2)	18 (7.8)	13 (9.6)	0.000	33 (9.9)	25 (10.1)	0.969
Agree	17 (8.5)	11 (3.0)		21 (9.6)	3 (1.3)	5 (3.7)		15 (4.4)	13 (5.3)	
Neither agree nor disagree	14 (7.0)	26 (7.0)		13 (5.9)	15 (6.5)	12 (8.9)		24 (7.1)	16 (6.5)	
Disagree	74(36.8)	129 (34.8)		84 (38.4)	79 (34.1)	44 (32.6)		118 (34.9)	90 (36.4)	
Strongly disagree	76 (37.8)	166 (44.7)		72 (32.9)	117 (50.4)	61 (45.2)		148 (43.8)	103 (41.7)	
**Q20. I would consider it reasonable to reduce the use of a mask in open areas**										
Strongly agree	48 (23.8)	65 (17.4)	0.224	47 (21.3)	39 (16.7)	29 (21.3)	0.228	76 (21.6)	39 (16.4)	0.290
Agree	51 (25.3)	92 (24.6)		49 (22.2)	56 (23.9)	42 (30.9)		92 (26.1)	53 (22.3)	
Neither agree nor disagree	52 (25.7)	92 (24.6)		51 (23.1)	67 (28.6)	31 (22.8)		82 (23.3)	66 (27.7)	
Disagree	32 (15.8)	78 (20.9)		45 (20.4)	44 (18.8)	26 (19.1)		65 (18.5)	50 (21.0)	
Strongly disagree	19 (9.4)	47 (12.6)		29 (13.1)	28 (12.0)	8 (5.9)		37 (10.5)	30 (12.6)	

Statistical significance (*p* < 0.05) was determined by a chi-square test. Abbreviations: Q, question.

**Table 4:** Use of personal protective measures after vaccination according to attitudes about the COVID-19 vaccine and intention of receiving COVID-19 vaccination.

If I Was Vaccinated…	Q8. Is the COVID-19 Vaccine the Most Effective Measure to Fight the Pandemic?			Q13. Would You Get the COVID-19 Vaccine?		
	No or Only in Combination with Other Protective Measures	Yes	*p*	No	Yes	*p*
**Q17. I would increase my social interactions in the short term**						
Strongly agree	26 (6.1)	27 (22.7)	0.000	7 (14.3)	38 (8.1)	0.394
Agree	94 (21.9)	40 (33.6)		12 (24.5)	115 (24.4)	
Neither agree nor disagree	167 (38.9)	32 (26.9)		18 (36.7)	171 (36.3)	
Disagree	118 (27.5)	16 (13.5)		8 (16.3)	121 (25.7)	
Strongly disagree	24 (5.6)	4 (3.4)		4 (8.2)	26 (5.5)	
**Q18. I would consider it reasonable to reduce the use of a mask with my regular contacts (friends, parents…)**						
Strongly agree	42 (9.5)	21 (19.4)	0.028	9 (17.7)	46 (9.8)	0.269
Agree	71 (16.0)	20 (18.5)		6 (11.8)	82 (17.4)	
Neither agree nor disagree	78 (17.6)	12 (11.1)		7 (13.7)	78 (16.5)	
Disagree	159 (35.8)	33 (30.6)		15 (29.4)	167 (35.4)	
Strongly disagree	94 (21.2)	22 (20.4)		14 (27.5)	99 (21.0)	
**Q19. I would consider it reasonable to reduce the use of a mask in closed public areas**						
Strongly agree	36 (8)	20 (20.2)	0.001	9 (18.8)	39 (8.2)	0.006
Agree	18 (4)	9 (9.1)		6 (12.5)	19 (4)	
Neither agree nor disagree	30 (6.7)	7 (7.1)		4 (8.3)	31 (6.5)	
Disagree	165 (36.7)	32 (32.3)		14 (29.2)	171 (36)	
Strongly disagree	201 (44.7)	31 (31.3)		15 (31.3)	215 (45.3)	
**Q20. I would consider it reasonable to reduce the use of a mask in open areas**						
Strongly agree	73 (16.1)	30 (30.3)	0.008	12 (26.1)	87 (18.1)	0.416
Agree	113 (24.9)	25 (25.3)		7 (15.2)	120 (24.9)	
Neither agree nor disagree	125 (27.6)	16 (16.2)		12 (26.1)	129 (26.8)	
Disagree	92 (20.3)	16 (16.2)		8 (17.4)	94 (19.5)	
Strongly disagree	50 (11.0)	12 (12.1)		7 (15.2)	54 (10.8)	

Statistical significance (*p* < 0.05) was determined by a chi-square test. Abbreviations: Q, question.

## Data Availability

The data presented in this study are available from the corresponding author (S.N.) upon reasonable request.

## Supplementary Materials

The following are available online at www.mdpi.com/article/10.3390/vaccines9101135/s1, Table S1: Questionnaire “Actitudes e intenciones hacia la primovacunación del SARS-CoV-2 en la población general”.
