# Supplementary material for: Attitudes and Intentions toward COVID-19 Vaccination among Spanish Adults: A Descriptive Cross-Sectional Study
Source: Vaccines (Basel). 2021 Oct 4;9(10):1135. doi: 10.3390/vaccines9101135 (PMC8538537; doi:10.3390/vaccines9101135)
Supplement: Supplementary file 1 [file vaccines-09-01135-s001.zip › vaccines-1377939-supplementary/written_permission_acknowledgement/Guadalupe Suarez.pdf]

El trabajador **Guadalupe Suarez Costa** ha colaborado de manera desinteresada para que la recogida de datos del estudio titulado " Attitudes and Intentions toward COVID-19 Vaccination Among Spanish Adults: A Descriptive Cross-Sectional Study" pudiera llevarse a cabo.

Para que así conste:

Fdo. Guadalupe Suarez Costa

Santiago de Compostela, 2 de octubre de 2021
