# Supplementary figures and images for: Attitudes and Intentions toward COVID-19 Vaccination among Spanish Adults: A Descriptive Cross-Sectional Study
Source: Vaccines (Basel). 2021 Oct 4;9(10):1135. doi: 10.3390/vaccines9101135 (PMC8538537; doi:10.3390/vaccines9101135)

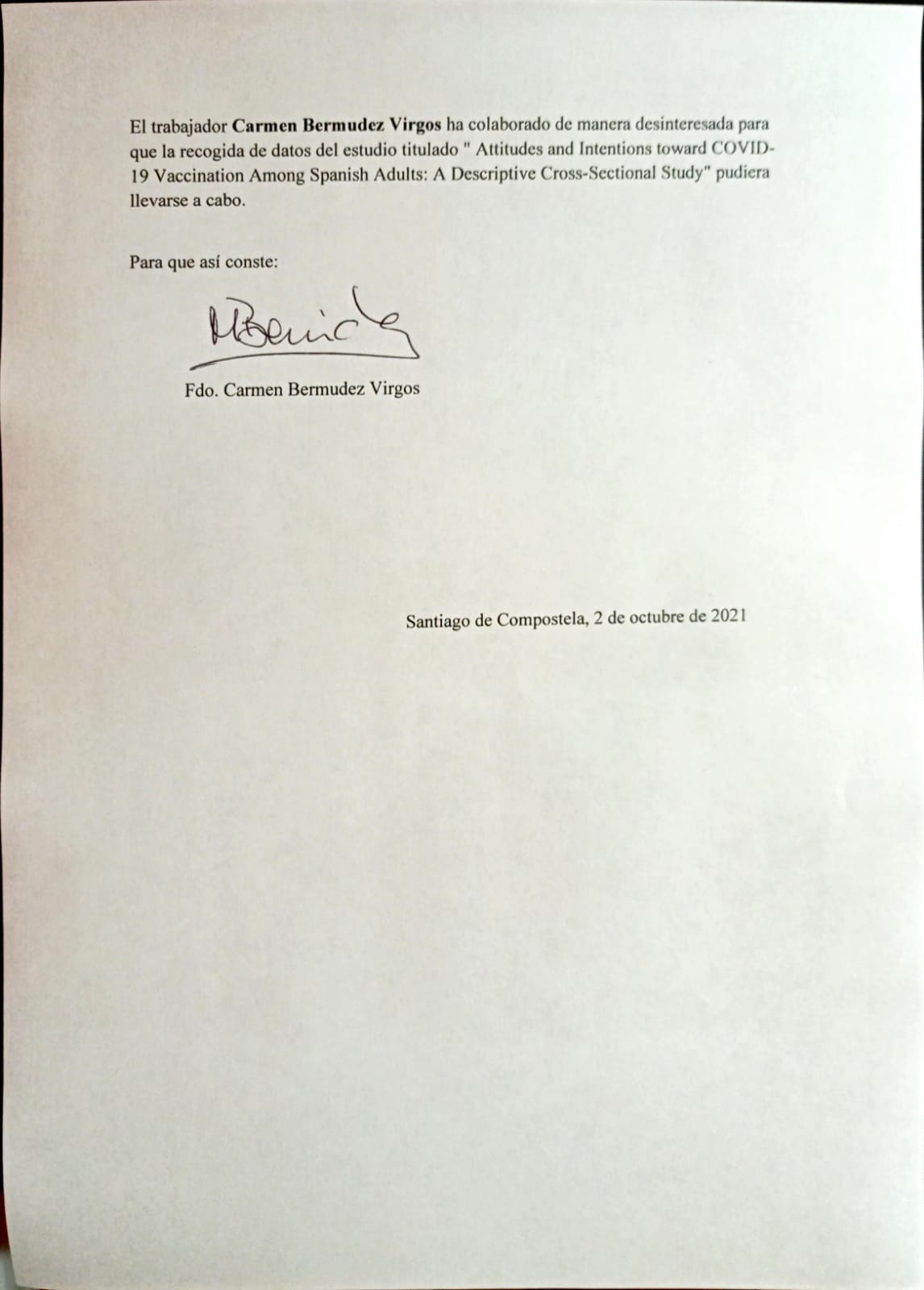

Supplement: Supplementary file 1 [file vaccines-09-01135-s001.zip › vaccines-1377939-supplementary/written_permission_acknowledgement/Carmen Bermudez.jpeg]

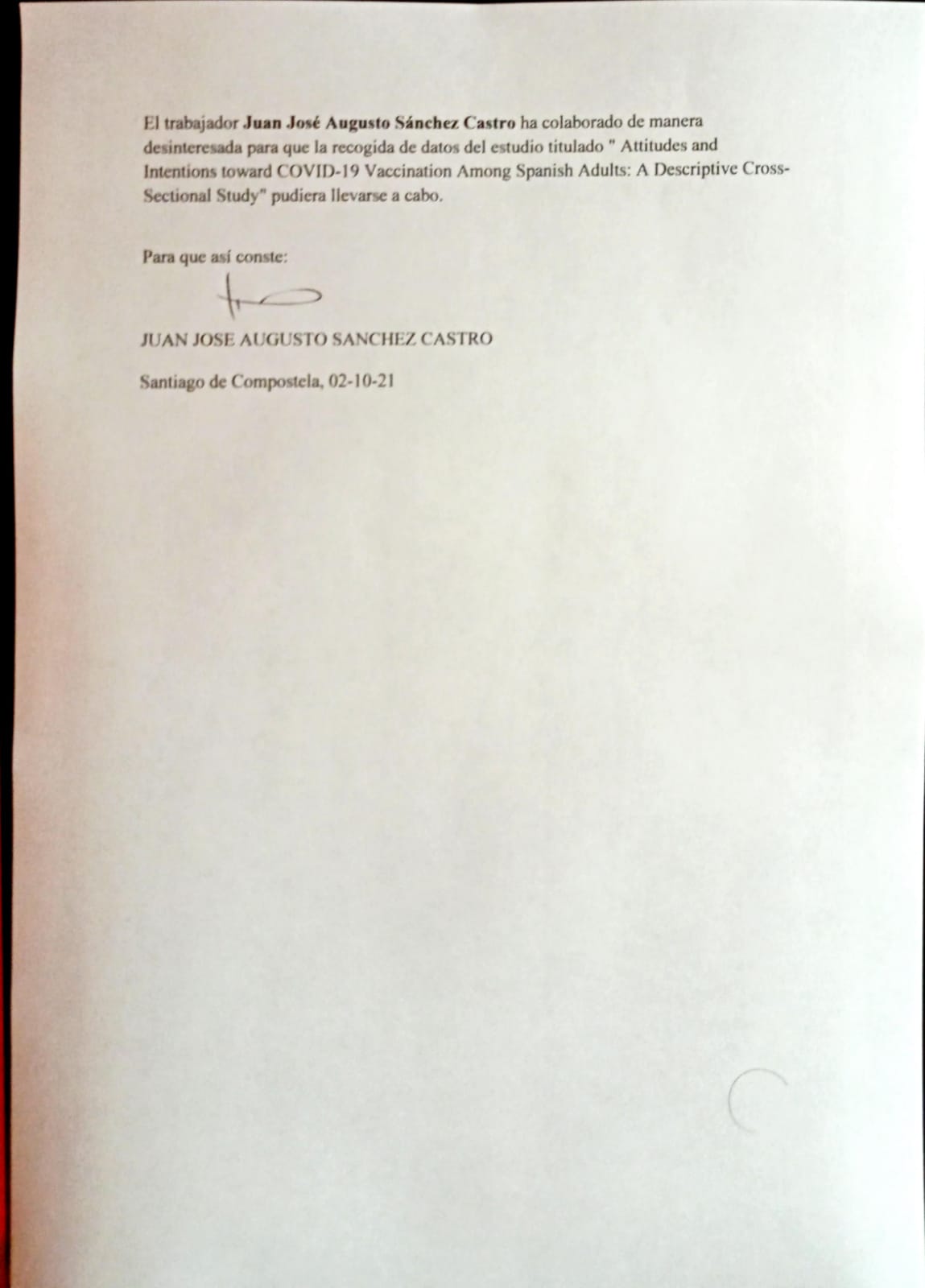

Supplement: Supplementary file 1 [file vaccines-09-01135-s001.zip › vaccines-1377939-supplementary/written_permission_acknowledgement/Juan Sanchez.jpeg]
